# Older Adults with Dementia Are Sedentary for Most of the Day

**DOI:** 10.1371/journal.pone.0152457

**Published:** 2016-03-31

**Authors:** Helena J. M. van Alphen, Karin M. Volkers, Christiaan G. Blankevoort, Erik J. A. Scherder, Tibor Hortobágyi, Marieke J. G. van Heuvelen

**Affiliations:** 1 Center for Human Movement Sciences, University Medical Center Groningen, Groningen, The Netherlands; 2 Department of Clinical Neuropsychology, VU University, Amsterdam, The Netherlands; 3 Lentis, Mental Health Care Institute, Zuidlaren, The Netherlands; University Of São Paulo, BRAZIL

## Abstract

**Purpose:**

Self-reported data suggest that older adults with dementia are inactive. The purpose of the present study was to objectively assess the physical activity (PA) levels of community-dwelling and institutionalized ambulatory patients with dementia, and to compare with the PA levels of cognitive healthy older adults.

**Methods:**

We used actigraphy to assess the PA levels in institutionalized (*n* = 83, age: 83.0 ± 7.6, Mini-Mental-State Examination (MMSE): 15.5 ± 6.5) and community-dwelling dementia patients (*n* = 37, age: 77.3 ± 5.6, MMSE-score: 20.8 ± 4.8), and healthy older adults (*n* = 26, age: 79.5 ± 5.6, MMSE-score: 28.2 ± 1.6). We characterized PA levels based on the raw data and classified <100 counts/min as sedentary behavior.

**Results:**

Institutionalized dementia patients had the lowest daily PA levels (1.69 ± 1.33 counts/day), spent 72.1% of the day sedentary, and were most active between 8:00 and 9:00 am. Institutionalized vs. community-dwelling dementia patients had 23.5% lower daily PA levels (difference M = 0.52, p = .004) and spent 9.3% longer in sedentariness (difference M = 1.47, p = .032). Community-dwelling dementia patients spent 66.0% of the day sedentary and were most active between 9:00 to 10:00 am with a second peak between 14:00 to 15:00. Community-dwelling dementia patients vs healthy older adults’ daily PA levels and sedentary time were 21.6% lower and 8.9% longer, respectively (difference M = 0.61, p = .007; difference M = 1.29, p = .078).

**Conclusions:**

Institutionalized and community-dwelling dementia patients are sedentary for most of the day and the little PA they perform is of lower intensity compared to their healthy peers. Their highest PA peak is when they get out of bed in the morning. In addition, it seems that institutionalized living is associated with lower PA levels in dementia patients. These are the first results that objectively characterize institutionalized as well as community-dwelling dementia patients’ PA levels and confirm that dementia patients are inactive.

## Introduction

Worldwide there is a general increase in the number of older adults and within this segment of the population, the number of individuals diagnosed with dementia is also rapidly increasing [1,2]. Considering the large costs and care burden associated with managing dementia, it is a health care priority to reduce the incidence of dementia, delay its onset, and minimize the adverse emotional, personal, and societal effects on patients, family members, peers, and care providers [3,4].

In recent years there has been a growing interest in PA as a non-pharmacological treatment for dementia [5]. Indeed, there is some evidence that regular PA can favorably affect dementia patients’ physical and cognitive function [6–8], quality of life [9], and activities of daily living (ADL’s) [7,10]. Conversely, physical inactivity and a sedentary lifestyle are associated with an increased risk for cardiovascular disease, metabolic aberrations, and other adverse health conditions [11] known to act as mediators in the early onset and progression of dementia [12].

Government guidelines, health authorities, and directives issued by the World Health Organization recommend that all adults engage for at least 30 minutes of moderate intensity PA (accumulated in bouts of at least 10 minutes) on five days or more per week [13–15]. Dementia patients are also encouraged to engage in regular PA and avoid an inactive lifestyle [13–15]. Becoming physically active from a sedentary state affords perhaps the most substantial favorable physiological changes, health benefits, and improvements in quality of life [9,16,17]. Therefore, there is a need to characterize dementia patients’ PA behavior in terms of duration, frequency, and intensity. Such information could then be used to design appropriate PA programs scaled specifically for dementia patients’ abilities, and to provide a basis for PA promotion in health care for this population.

Although the feasibility of objectively assessing PA in a sample of dementia patients living in the community has been demonstrated (*n* = 26) [18], previous studies characterized dementia patients’ PA levels and patterns based on self-report [19–23]. The emerging picture is that the quantity and intensity level of dementia patients’ PA are low compared with healthy controls [19–23]. A previous study, for example, reported a 39.7% lower score on the Physical Activity Scale of the Elderly (PASE) in a sample of patients with dementia compared to age-matched dementia-free controls [21]. In addition, primarily unstructured and low-intensity activities for dementia patients were reported in studies using subjective reports and questionnaires, e.g., the Baecke Questionnaire Modified for the Elderly (BQME) [22,23].

Based on self-report it is also indicated that the type of dementia relates to different degrees of activity. A previous study using an extension of the Nottingham Activity Scale reported higher activity levels for patients diagnosed with Alzheimer’s disease (AD) compared to patients diagnosed with Parkinson’s disease dementia, dementia with Lewy bodies, or vascular dementia [19].

Dementia patients’ PA levels and patterns reported so far may not be reliable and representative because self-reported vs. objective measures are less accurate to quantify PA and are especially unsuitable to gauge PA intensity [24] and walking behavior [25]. In addition, most dementia patients themselves are not capable to recall or report their PA behavior and self-reported PA measures are inadequate to reconstruct 24 h PA patterns. While it cannot identify the type of PA performed, Actigraph, a wrist-worn accelerometer, can provide objective and continuous information about the duration, frequency, and intensity of PA and also about sedentary time with considerable precision.

Although previous studies reported the PA levels in dementia patients [19–23, 26], most studies did not measure sedentary behavior or differentiated it from light activity intensity. In addition, little is known about the impact of institutionalized living on the PA levels of patients with dementia and most studies did not compare PA levels among different types of dementia. Taken together, the purpose of the present study was to objectively quantify and characterize the daily PA levels and patterns of ambulatory patients with dementia who regularly visit a day care center or live in a care facility. The main analyses compared PA levels between community-dwelling and institutionalized dementia patients and also compared their PA levels to the PA levels in health older adults. In addition, the analyses compared PA levels among different types of dementia.

## Methods

### Participants

Dementia patients in the present study were enrolled in a longitudinal study that examined the effects of regular PA on health [27]. In this longitudinal study, participants were recruited via medical staff of aged care facilities. First, the medical staff was informed about the goal and procedure of the study. Secondly, possible participants were selected within subunits of the institutions by a team, including the researcher, medical staff and nurses. Third, an information letter with informed consent was sent to the legal representatives of the participants, together with an invitation to attend an oral presentation [27]. Based on a sample size calculation 175 participants had to be included [27], which was possible with 13 nursing homes (*n* = 130) and 4 four daycare centers (*n* = 45) located in the Netherlands. The participants or their legal guardians gave oral and written informed consent prior to enrollment. The Medical Ethical Committee of the VU University of Medical Center approved the study protocol.

We performed secondary analyses on the baseline measurements of the abovementioned study [27]. We only included participants with 1) a diagnosis of dementia in their medical record, 2) being ambulatory with or without a walking aid (walker, cane), and 3) actigraphy data for at least six consecutive days, 24 h per day. Twenty participants were excluded because of no or missing dementia diagnosis from the medical records. Another 35 participants were excluded due to missing or insufficient actigraphy data. More specifically, the PA measurement failed in 22 participants (participants refused to wear an accelerometer (*n* = 11), temporarily impossible (*n* = 4), technical problems (*n* = 4), Actiwatch was lost (*n* = 2), organizational problems (*n* = 1)), and the data in 13 participants were gathered less than six consecutive days, 24 h per day. Therefore, the current study is based on data in 120 dementia patients (83 institutionalized, 37 living in the community) from 13 different nursing homes and 4 four daycare centers.

To compare dementia patients’ PA levels with those of cognitive healthy older adults we used data from cognitive healthy spouses of institutionalized dementia patients (n = 35). These partners lived independently in their own homes, while their spouses lived in an institution. Inclusion criteria were: 1) a partner with a diagnosis of dementia in their medical record, 2) cognitive healthy (MSSE > 23), 3) being ambulatory with or without a walking aid (walker, cane), and 4) actigraphy data for at least six consecutive days, 24 h per day. Nine participants were excluded due to missing or insufficient actigraphy data. Therefore the current study consisted of 26 cognitively healthy older adults.

Table 1 shows the characteristics of the final sample. Institutionalized dementia patients vs. community-dwelling dementia patients were older (t (118) = 3.856, p < .001), used more frequently a walking aid or cane (Chi^2^ = 5.882, p < .053), had lower Mini-Mental-State Examination (MMSE) [28] scores (t (111) = 4.318, p < .001), and more of them were female (Chi^2^ = 17.723, p < .001). Community-dwelling dementia patients vs. healthy older adults had lower MMSE-scores (t (44.461) = 8.492, p < .001), were slightly younger (t (61) = 1.507, p = .137), used somewhat more frequently a walking aid or cane (Chi^2^ = 1.076, p = .300), and more of them were female (Chi^2^ = .553, p = .457).

**Table 1:** Participant characteristics of 83 institutionalized and 37 community-dwelling dementia patients, and 26 healthy older adults.

	Institutionalized dementia patients (*n* = 83)	Community-dwelling dementia patients (*n* = 37)	Healthy older adults (*n* = 26)
*Characteristics*	Mean (SD)/n (%)	Mean (SD)/n (%)	Mean (SD)/n (%)
Age (years)	83.0 (7.6)	77.3 (5.6)	79.5 (5.6)
BMI (kg/m^2^)^1^	26.9 (4.9)	26.6 (3.4)	n.a.
MMSE (0–30)^2^	15.5 (6.5)	20.8 (4.8)	28.2 (1.6)
Gender (% women)	79.5	40.5	50.0
Walking and Mobility (% aid users)	42.7	12.5	4.3
Diagnosis:			
Alzheimer’s Disease (%)	49.4	48.6	
Vascular dementia (%)	14.5	16.2	
Alzheimer with vascular problems (%)	9.6	10.8	
Dementia with Lewy bodies (%)	0.0	8.1	
Frontotemporal dementia (%)	6.0	2.7	
Parkinson’s disease dementia (%)	0.0	5.4	
Korsakoff dementia (%)	2.4	2.7	
Dementia type not specified (%)	18.1	5.4	

^1^BMI = Body Mass Index

^2^MMSE = Mini-Mental State Examination

### Assessment of Physical Activity

Participants wore an Actiwatch (AW-4, Cambridge Neurotechnology Ltd., Cambridge, UK) on their dominant wrist that recorded wrist accelerations. The instrument contains an acceleration-responsive piezoelectric sensor and is set up to record the integration of intensity, amount and duration of movement in three directions (most sensitive in the vertical axis; omnidirectional). The acceleration signal is digitally sampled at 32 Hz so that an increase in speed and motion produces an increase in voltage. The corresponding voltage, i.e., the peak amplitude of movement acceleration is integrated and stored as an activity count in the Actiwatch memory. These activity counts are summed across an operator-defined time interval (the epoch length). The count is quantified as 0 within an epoch for an activity period without accelerations. Each participant wore one Actiwatch for a minimum of six consecutive days, 24 h per day, as these instruments are waterproof. We used an epoch duration of 60 s, which yields 1440 epochs per day.

We evaluated the raw data, which represented the PA level without imposing cut-point decisions. The amount of PA for each participant was quantified by the time spent in specific zones of activity counts with ranges of 100 counts/min. We also classified <100 counts/min as sedentary behavior [29], and calculated the total daily activity, which is the sum of all counts per 24 h. All counts were divided by 100,000 to facilitate the presentation and interpretation of the results.

### Statistical Analysis

Total daily PA and hourly mean PA counts per minute were not normally distributed, thus log-transformed (log^10^) data were used in the statistical analyses. Independent *t*-tests were used to compare the total daily PA levels and time spent in sedentariness per group between men and women. One-way ANOVA with post-hoc tests (Games-Howell, because of unequal variances) was used to compare outcome variables between institutionalized patients with dementia, community-dwelling patients with dementia, and healthy older adults. In addition, two-way ANCOVA was used to analyze the differences in the outcome variables between the institutionalized patients with dementia, community-dwelling patients with dementia, and healthy older adults, with adjustments for potential confounders: age, and cognitive state (MMSE) were included as covariates, use of a walking aid or cane was included as an additional fixed factor (main effect only). Two-way ANOVA was used to compare the outcome variables of patients diagnosed with AD, and patients with other dementia types in interaction with living situation (institutionalized vs. community based). One-way ANOVA with post-hoc tests (Bonferroni) was used to compare the number of minutes spent in specific zones of activity counts and the hourly mean PA counts per minute between the institutionalized patients with dementia, community-dwelling patients with dementia, and healthy older adults. P-values lower than 5% were considered to indicate statistical significance.

## Results

### Total Daily PA Levels

In none of the three groups total daily PA was significantly related to gender. Therefore, the results are presented for men and women together. Total daily PA levels were different for the three groups (F (2,143) = 14.477, p < .001). Post-hoc analyses showed that the total daily PA levels of institutionalized dementia patients were 23.5% lower than those of community-dwelling dementia patients (difference M = 0.52, p = .004), and 40% lower than those of healthy older adults (difference M = 1.13, p < .001). Community-dwelling dementia patients vs. healthy older adults had 21.6% lower daily PA levels (difference M = 0.61, p = .007) (Table 2). After controlling for potential confounders (age, MMSE and use of walking aid) the differences between the three groups remained significant (F (2,120) = 4.274, p = .016). In addition, there was a significant effect of the use of a walking aid on the total daily PA levels (F (1,120) = 6.328, p = .013). People using a walking vs. people not using a walking aid had 37.2% lower total daily PA levels. In S1A Fig, total daily PA levels are presented per group stratified for the use of a walking aid.

**Table 2:** Total daily PA and sedentary time for institutionalized and community-dwelling dementia patients and healthy older adults.

		Total daily PA (counts/day)^a^^,^^b^^,^^c^^,^^d^	Sedentary behavior (h/day)^a^^,^^e^
Institutionalized	All (n = 83)	1.69 (1.33)	17.30 (3.24)
	Alzheimer’s disease (n = 49)^f^	1.86 (1.54)	16.82 (3.43)
	Other dementia types (n = 34)	1.45 (0.93)	18.0 (2.85)
Community- dwelling	All (n = 37)	2.21 (1.26)	15.83 (2.72)
	Alzheimer’s disease (n = 22)^f^	2.38 (1.11)	15.83 (2.65)
	Other dementia types (n = 15)	1.97 (1.44)	16.60 (2.73)
Healthy control	All (n = 26)	2.82 (0.83)	14.54 (1.92)

^a^ Mean (SD)

^b^ values x 10^5^

^c^ Total daily PA is the sum of activity counts per 24 h

^d^ Statistical tests based on log transformed data, but actual means included for ease of interpretation

^e^ Averaged sedentary h/day based on cut-point <100.

^f^ Alzheimer’s disease with or without vascular problems

To compare patients diagnosed with AD (without or with vascular problems) and non-AD patients we did not find a significant difference in total daily PA between these two groups (F (1, 116) = 2.955, p = .088), although the AD patients vs. the non-AD patients were 25.5% more active. There was no interaction effect between living situation (community vs. institution) and dementia type (AD vs. other dementia types) (F (1,116) = .027, p = .870). However, the difference in daily PA levels between institutionalized and community-dwelling patients remained significant (F (1,116) = 7.920, p = .006). Institutionalized vs. community-dwelling patients with AD had 21.8% lower total daily PA levels and institutionalized vs. community-dwelling patients without AD had 26.4% lower total daily PA levels (Table 2). In S2A Fig, total daily PA levels are presented for each dementia diagnosis separately.

### Sedentary time

In none of the three groups sedentary time was significantly related to gender. Therefore, the results are presented for men and women together. The time spent in a sedentary state was different for the three groups (F (2,143) = 9.891, p < .001). Post-hoc analyses revealed that institutionalized dementia patients spent 9.3% more time in a sedentary state compared to community-dwelling dementia patients (difference M = 1.47, p = .032) and 19.0% more time compared to healthy older adults (difference M = 2.76, p < .001) (Table 2). Community-dwelling dementia patients vs. healthy older adults spent 8.9% more time in a sedentary state, but the mean difference was non-significant (difference M = 1.29, p = .078) (Table 2). After controlling for potential confounders (age, MMSE and use of walking aid) the differences between the three groups remained significant (F (2,120) = 3.518, p = .033). In addition, there was a significant effect of the use of a walking aid on sedentary time (F (1,120) = 4.145, p = .044). People using a walking vs. people not using a walking aid spent 12.4% more time in a sedentary state. In S1B Fig, time spent in a sedentary state is presented per group stratified for the use of a walking aid.

To compare patients diagnosed with AD (without or with vascular problems) and non-AD patients we found a significant difference in sedentary time between these two groups (F (1, 116) = 4.013, p = .047). AD patients vs. the non-AD patients spent 6.9% less time in a sedentary state (Table 2). There was no interaction effect between living situation (community vs. institution) and dementia type (AD vs. other dementia types) (F (1,116) = .008, p = .929). However, the difference in sedentary time between institutionalized and community-dwelling patients remained significant (F (1,116) = 5.572, p = .020). Institutionalized vs. community-dwelling patients with AD spent 9.8% more time in a sedentary state and institutionalized vs. community-dwelling patients without AD spent 6.3% more time sedentary. In S2B Fig, time spent in sedentary state is presented for each dementia diagnosis separately.

### Daily PA Patterns

**Fig 1:**
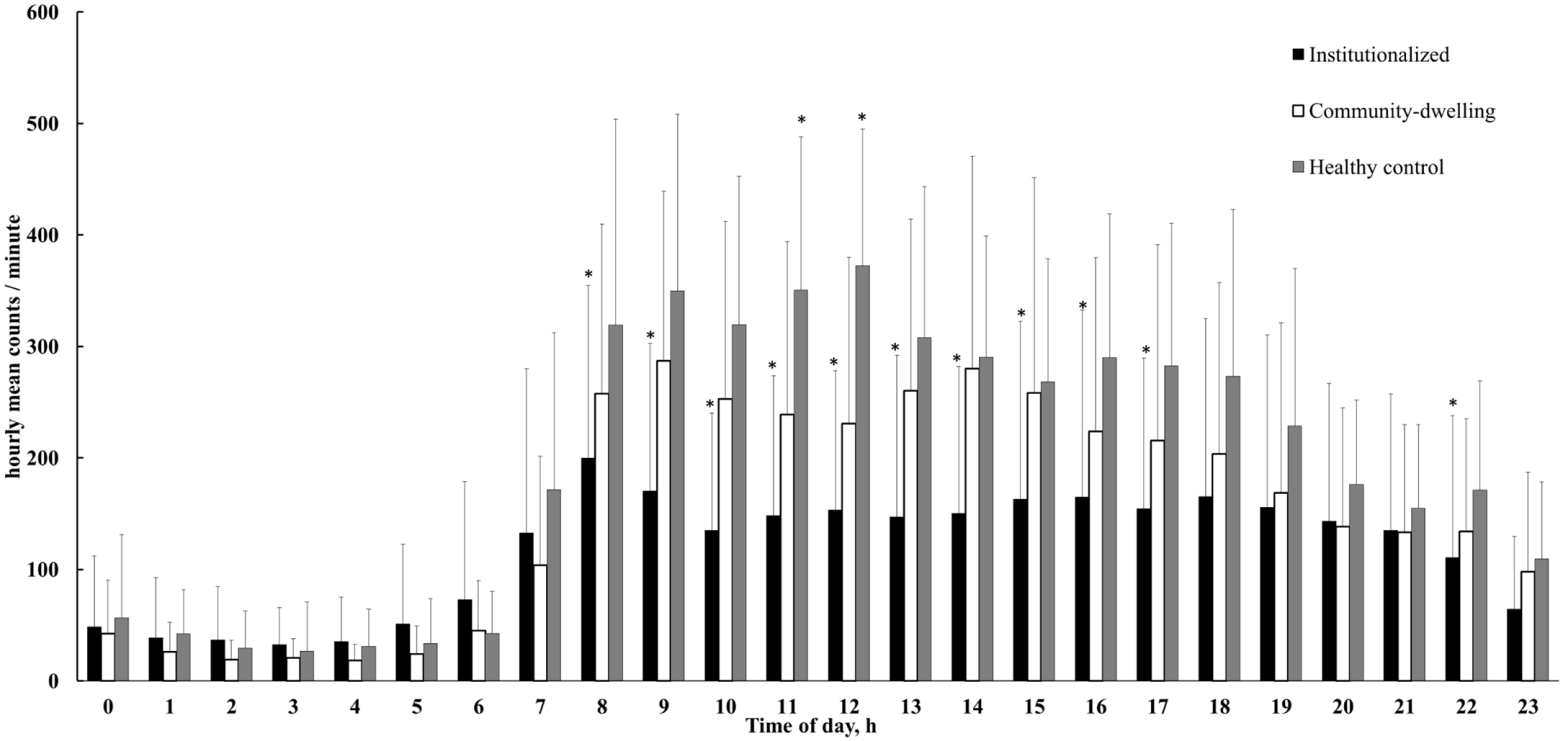
Daily physical activity (PA) pattern in older adults with and without dementia. The figure shows the hourly PA during the day based on minute-averaged data, starting at midnight (0). The first bars show the hourly mean counts/minute from 0:00 to 1:00 h. Filled bars: dementia patients living in an institution. Open bars: community-dwelling dementia patients. Gray bars: community-dwelling healthy older adults. Values are mean (and standard deviation). *, different to community-dwelling dementia patients (p < 0.05).

Fig 1 shows the pattern of hourly mean PA counts per minute. Institutionalized vs. community-dwelling dementia patients were significantly less active from 8:00 to 18:00 h, and from 22:00 to 23.00 h. For daytime activity (7:00 to 23:00 h), there was one small peak activity for institutionalized dementia patients between 8:00 and 9:00 h, while community-dwelling dementia patients were most active between 9:00 to 10:00, as well as 14:00 to 15:00.

Healthy older adults were most active between 9:00 to 10:00 h and between 11:00 to 13:00 h. Comparing community-dwelling dementia patients and healthy older adults, we found that community-dwelling dementia patients were significantly less active than healthy older adults from 11:00 to 13:00 h.

### Range of PA Intensity

**Fig 2:**
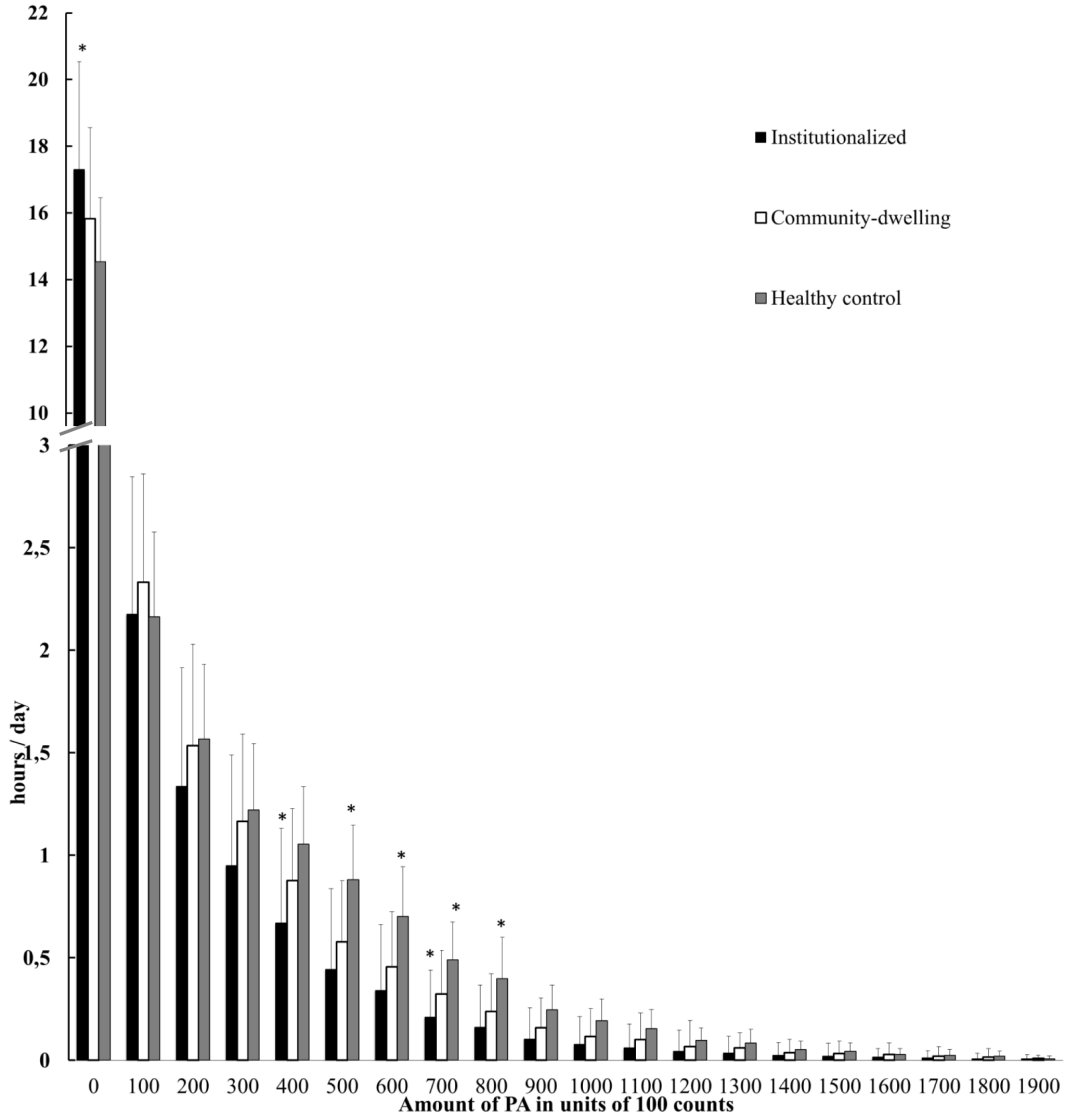
Quantity of physical activity (PA) as inferred from the time spent in specific activity count zones. PA counts were binned in units of 100 counts. The first bars show the daily hours spent in sedentary time (0–99 counts). Note the axis interruption that highlights the large number of hours spent in sedentary time vs. other activity counts zones. Filled bars: dementia patients living in an institution. Open bars: community-dwelling dementia patients. Gray bars: community-dwelling healthy older adults. Values are mean (and standard deviation). *, different to community-dwelling dementia patients (p < 0.05).

Fig 2 shows the amount of PA as interfered from the number of minutes spent in specific zones of activity counts. Institutionalized dementia patients did spent 72.1% of the day in a sedentary state (0 to 99 counts/min) (17.3 ± 3.2 h) (Fig 2). The proportion of activity performed above the upper bound of sedentary behavior (99 counts/min) was significantly lower in institutionalized compared to community-dwelling patients with dementia (difference M = 86.75, p = .030). For both groups there was minimal activity above 2,000 activity counts/min (<0.05% per day). Both patient groups showed a similar PA pattern with a greater proportion of PA performed at low intensity levels (F 2). However, institutionalized compared to community-dwelling patients spent significantly less time between 0 and 99 counts/min, 400 and 499 counts/min, and 700to 799 counts/min.

Healthy older adults showed a similar daily PA pattern as community-dwelling patients with dementia. Both groups performed a greater proportion of activity at lower intensity levels and minimal activity above 2,000 activity counts/min (<0.05% per day). However, healthy older adults spent significantly more time in higher activity ranges. Specifically, healthy older adults spent significantly more time between 500 and 900 counts/min compared to community-dwelling patients with dementia.

## Discussion

The main finding of the present study is that institutionalized as well as community-dwelling patients with dementia are sedentary for most of the day and they perform their little PA at a lower intensity level than their healthy peers. For institutionalized as well as community-dwelling patients with dementia the highest within-day PA peak occurred around the morning waking hours between 8:00 and 10:00 am, with a second peak for community-dwelling patients between 14:00 and 15:00. Institutionalized vs. community-dwelling dementia patients had 23.5% lower total daily PA and were 9.3% longer sedentary. In comparison with healthy older adults, community-dwelling dementia patients’ total daily PA levels and sedentary time were 21.6% lower and 8.9% longer, respectively. AD patients vs. non-AD patients spent 6.9% less time sedentary. We discuss these findings in the context of the PA measurements, daily levels and patterns of PA, PA levels among different types of dementia, and PA prescriptions for dementia patients.

### Measurements of PA

The current study quantified dementia patients’ daily PA levels, characterized their PA patterns, and derived estimates of relative PA intensity based on actigraphy. This method appeared feasible in patients with dementia since only 20% of the current sample had to be excluded because of missing or insufficient actigraphy data (fewer than six days of valid data). A comparable success rate was achieved in other studies in people without dementia [30,31]. Based on a previous study in healthy older adults six days of valid data can be considered as representative for the weekly PA behavior [32]. Three days of accelerometer data are needed to predict PA levels [32,33] and five days of complete data to reliably predict total time spent in sedentary behavior [32].

### Daily PA levels

Comparing the PA of current community sample with dementia with a similar dementia group, the total daily PA levels in current study (2.21 counts/day) compare well with those reported previously (2.11 counts/day) [26]. The total daily PA levels of current healthy sample (2.82 counts/day) were slightly (8.1%) lower compared to a similar dementia-free sample (3.07 count/day), probably due to the low sample size in current study (n = 26) vs. this previous study (n = 624).

Current study found lower PA levels for institutionalized compared to community-dwelling dementia patients. This finding was consistent among different diagnoses (except for a frontotemporal dementia diagnosis based on *n* = 1) and is in line with another study showing shorter daily standing and stepping times and longer sitting/lying times for older adults living in residential care compared to older adults living in the community (sitting/lying: >12 h/day vs. 8.4–11.0 h/day, respectively) [31]. Therefore, it seems that institutionalized living is associated with lower PA levels in patients with dementia.

The lower PA levels of institutionalized dementia patients vs. community-dwelling patients could be partly explained by older age, more severe cognitive impairments and more frequent walking aid use. Previous studies using accelerometer-based measurements in dementia-free older adults also revealed an age effect [30,34]. For example, a 70–75 year age group had more than three times higher activity counts per minute compared with the ≥85 year age group (237.8 vs. 75.4 counts/min) [31]. A recent review of PA-correlates in a community-dwelling dementia sample showed that physical factors as global motor function and gait speed were found to be positively associated with PA, but not lower global cognition [35]. Nevertheless, even after adjustment for age, global cognitive function (MMSE) and walking aid use, institutionalized vs. community-dwelling dementia patients had significantly lower total daily PA levels. So, other factors will also play a role here such as differences in physical environment and (absence of) PA policies in care institutions. Future research will have to characterize more comprehensively the influence of the physical environment and PA policies since these factors are understudied [35]. Active PA policies and the support of caregivers within care institutions may facilitate the PA behavior of institutionalized patients. However, the (absence of) PA policies in institutions are not inventoried in this study. ‘Activating care’ is a recent development and not fully implemented yet. Our results suggest that a lot of work needs to be done here. The main PA peak in the early morning of institutionalized dementia patients seems to be related to their basic activities of daily living instead of organized activities. Future research on the effectiveness of PA policies in care institutions is warranted.

In current study lower total daily PA levels in community-dwelling patients vs. healthy peers were found. This finding is in line with previous studies showing lower total daily PA levels for participants with dementia vs. participants without dementia [19–23, 26]. In addition, community-dwelling dementia patients spent significantly less time at higher relative physical activity intensities (less minutes in higher activity count zones), so the few activities they performed were at lower intensity. The PA levels of community-dwelling dementia patients who regularly visit a daycare center could be influenced by the PA policy and routine of those daycare centers. However, we did not study the (absence of) PA policies in daycare centers. Considering the PA peak in the morning and another PA peak in the afternoon around 14:00–15:00 h, it is possible that this second peak is due to organized activities. However, future research on the effectiveness of PA policies in daycare centers is recommended.

### Daily PA patterns

This study is the first study that provides an objective insight into the 24 h patterns of patients with dementia. Institutionalized compared to community-dwelling dementia patients were less active during the day with most marked differences between 8:00 and 19:00 h (77.4 counts/min). In the evening (19:00 to 23:00 h) the differences were small (12.6 counts/min). Previous studies reported that age differences in PA patterns were prominent in the evening hours [30], however, differences in PA between institutionalized and community-dwelling dementia patients were most marked in the daytime hours. Therefore, the daytime activity makes a major contribution to the differences in total daily PA between community-dwelling and institutionalized dementia patients. Considering the PA peak in the morning for institutionalized and community-dwelling dementia patients and another PA peak for community-dwelling patients in the afternoon around 14:00–15:00 h, future studies should determine the optimal time when dementia patients should receive PA stimulus. There could be an interaction between the living situation and the optimal timing of daily PA stimulus. Since institutionalized (42.7%) vs. community-dwelling dementia patients (32.1%) used 3.5 times more a walking aid or cane, with the institutionalized aid-users vs non aid-users performing 32.1% less PA, we suggest that participants with lower physical capacity as explained by the use of a walking aid need more stimuli for PA. However, they are also more frail and simply unable to favorably respond to a nudge for PA, especially if this prompt comes in the morning hours, because daily chores in the morning consume most of these patients’ energy. In total, additional studies are needed to verify our results to identify optimal timed patterns with a special reference to the living situation and patients’ maximal physical capacity, and to determine how other factors not examined here such as gender could affect the timing of the PA stimulus.

### PA levels among different types of dementia

Although the low sample sizes for individual diagnoses (other than AD) did not allow for statistical testing, the present study showed slightly higher total daily PA levels and lower sedentary time for patients diagnosed with AD compared to non-AD patients. This is in line with a previous study showing higher activity levels for patients diagnosed with AD compared to patients diagnosed with Parkinson’s disease dementia, dementia with Lewy bodies, or vascular dementia [19]. However, the present study indicates that not a vascular dementia diagnoses, but the less common dementia diagnoses such as dementia with Lewy bodies (*n* = 3), frontotemporal dementia (*n* = 6), Parkinson’s disease dementia (*n* = 2), and Korsakoff dementia (*n* = 3) account for the differences in PA between AD and non-AD patients. However, future research with larger sample sizes is needed to investigate the different degrees and patterns of PA among different types of dementia.

### PA prescriptions

For health benefits, government guidelines recommend 30 minutes of moderate intensity PA accumulated in at least 10-minute bouts on five or more days per week [13–15]. Specific cut-points used to convert activity counts into energy expenditure or METs dramatically affect PA compliance with health recommendations [36,37]. In addition, these contentious cut-points for moderate-to-vigorous PA (MVPA) are mostly derived from a device worn at the hip [36–38], while in the present study subjects wore the Actiwatch on the wrist. Unfortunately, previous studies showed that data comparisons between hip and wrist derived data are inappropriate [38–40]. Activity counts derived from the wrist vs. the hip are systematically higher [39,40]. In addition, activity counts are not comparable across different models of accelerometers [39]. Altogether, we were not able to quantify the time spent in MVPA (absolute intensity), but we were able to describe the amount of activity within different zones of activity counts (relative intensity). We found that institutionalized dementia patients spent 72% of the day between 0 and 100 activity counts/min, which we and others [29] defined as sedentary time. Such an activity count probably still underestimates sedentary time because in the previous study that used the same cut-point for sedentary behavior the subjects wear an Actiwatch on the hip [29]. The alarmingly long time spent in a sedentary state by dementia patients is an important finding because participation in high levels of MVPA cannot fully mitigate the health risks associated with prolonged sedentary time [41]. Thus, even with low cut-points for moderate intensity PA, dementia patients would still be qualified as highly sedentary, a state associated with an increased risk for cardiovascular disease, metabolic aberrations, and other adverse health conditions [11], accelerating the progression of dementia [12]. Until disease-specific objective PA recommendations are established, our recommendation for PA prescription is based on the concept that dementia patients would benefit from even small reductions in sedentary time because becoming physically active from a sedentary state affords perhaps the most substantial favorable physiological changes and health benefits [16,17]. One way to reduce sedentary time is by interrupting prolonged periods of sedentariness using PA rather than performing more PA at a higher intensity at times of the day when dementia patients are already active. Especially for institutionalized patients our advice is to stimulate PA in the afternoon, because we suggest that daily chores in the morning as interpret by their highest PA peak consume most of these patients’ energy.

### Limitations

One limitation is that we assumed that all activity counts per minute represent the same PA intensity for healthy as well as demented older adults, younger as well as older dementia patients, and aid-users as well as non-aid users. However, altered gait patterns and an increased metabolic cost for activities in older adults with dementia have been reported [42,43]. Further calibration of activity counts per minute may be required for this specific patient population. In addition, an expectation is that a wrist-worn device would underestimate PA in aid-users. However, we note that even after an adjustment for the use of a walking aid, PA levels were lower and sedentary times were higher in community-dwelling dementia patients vs. healthy older adults, and institutionalized patients vs. community-dwelling patients.

Another limitation is related to the selection procedure of institutions and daycare centers in the present study: homes were approached based on existing collaboration with the VU university. Small differences between homes as a result of for instance a different location, materials, PA policy, and efforts of family or volunteers, may have influenced our results. However, we think that the homes and daycare centers in the present study are representative because the elderly care is organized at national level and homes and daycare centers have to meet strict national laws and rules with respect to care and organization. In addition, all dementia patients were diagnosed with dementia by the national ‘care indication center’(CIZ), whose diagnosis and referral are mandatory in order to gain access to special geriatric care in the Netherlands.

Another point to address is that our healthy control sample consisted of spouses of institutionalized patients with dementia. A first thought might be that this is a very specific group. However, dementia is a disease that can affect all, despite of social economic state, education and other factors. Therefore, we do not think generalizability is hampered. Even more, people who volunteer to participate as a control group in studies about PA are in most cases relatively healthy. Because we actively invited spouses of patients it might even that we obtained a more genuine control group which enhances the generalizability of our control group. However, the slightly lower PA levels for current healthy sample vs. a similar healthy sample [26] could be the result of an underestimation of healthy older adults’ PA levels as a direct result of the time and care burden related to a dementia partner.

Another limitation is that local geography, environmental conditions, and the nature of health care itself can affect PA data so that caution is needed to compare PA data between pools of dementia patients living in different countries.

Finally, current study made no distinction between weekdays and weekend days and between different seasons. However, PA levels of retired dementia patients differ little for the different days of the week due to a rather fixed daily schedule. In addition, we note that the highest PA peak is when they go out of bed in the morning, which occurs on a daily basis. However, it is possible that the PA levels of community-dwelling patients differ between the days of the week they attend (usually two and maximal three times per week) vs. not attend a daycare program. Since the higher PA levels for community-dwelling patients in the afternoon may be due to organized activities at daycare centers it is possible that using averaged PA levels may have overestimated the PA levels on days at home and underestimated the PA levels for days attending a daycare program. However, despite this issue the weekly time spent in a sedentary state even remains alarmingly long. In addition, a previous study noted that the regular PA patterns in relatively healthy older adults (*n* = 230, mean age: 78.1) appeared to be largely uninterrupted by seasonal differences [34]. Therefore, we do not expect that six instead of seven days of valid activity data for almost half of the participants or seasonal influences biased our results.

## Conclusion

Dementia patients are sedentary for most of the day and the little PA they perform is of lower intensity compared to their healthy peers. The highest PA peak is when they get out of bed in the morning. In addition, it seems that institutionalized living is associated with lower PA levels in dementia patients. These are the first results that objectively characterize institutionalized as well as community-dwelling dementia patients’ PA levels and confirm that dementia patients are inactive and emphasize the importance of PA policies to promote PA in patients with dementia.

## Supporting Information

S1 Fig PA levels stratified for the use of a walking aid. (A) Total daily PA levels. (B) Time spent in a sedentary state.
(TIF)

S2 Fig PA levels among different types of dementia. (A) Total daily PA levels. (B) Time spent in a sedentary state.
(TIF)

S1 File Dataset. (SAV)
