# Supplementary figures and images for: Older Adults with Dementia Are Sedentary for Most of the Day
Source: PLoS One. 2016 Mar 31;11(3):e0152457. doi: 10.1371/journal.pone.0152457 (PMC4816298; doi:10.1371/journal.pone.0152457)

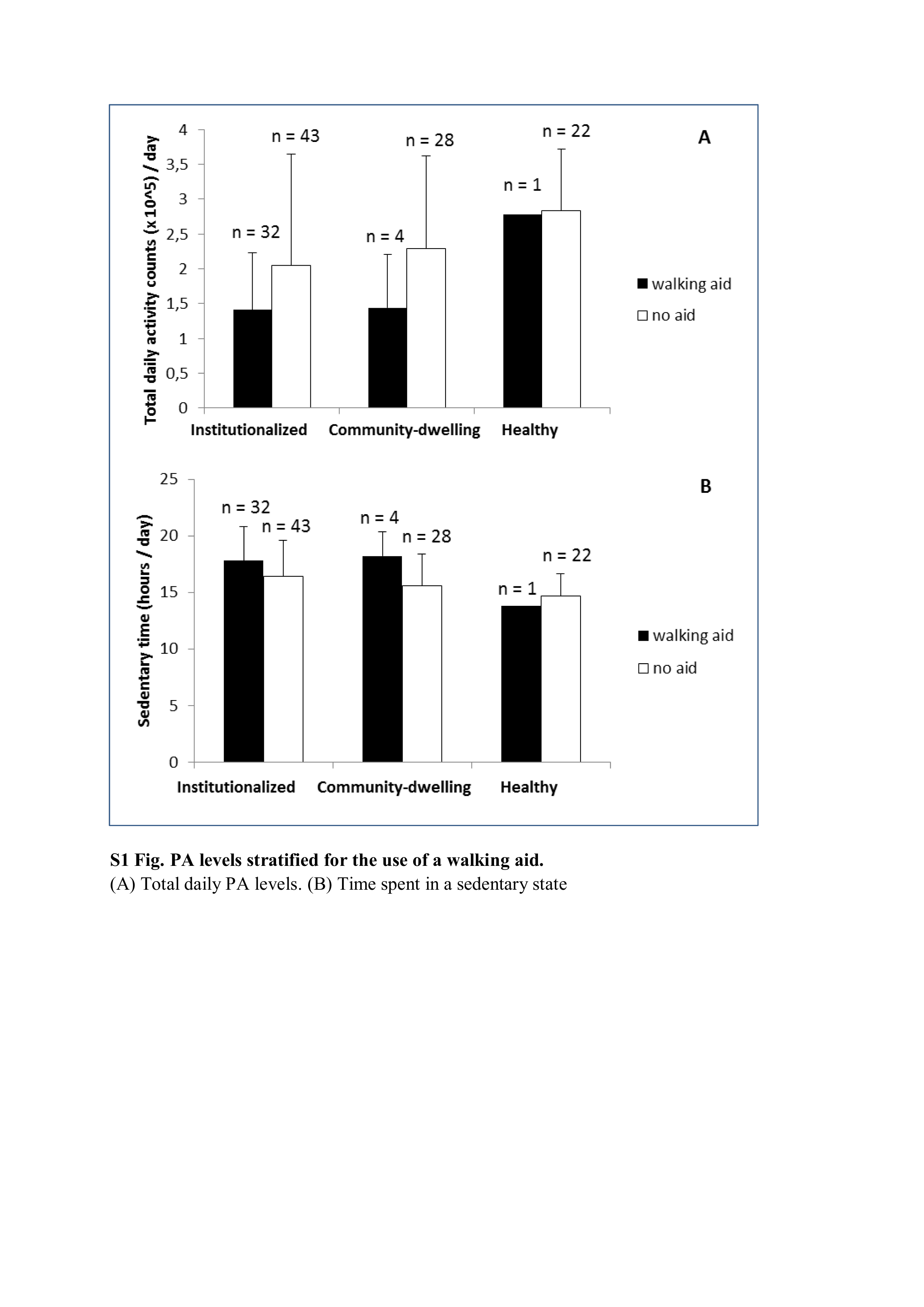

Supplement: S1 Fig — (A) Total daily PA levels. (B) Time spent in a sedentary state. (TIF) [file pone.0152457.s001.tif]

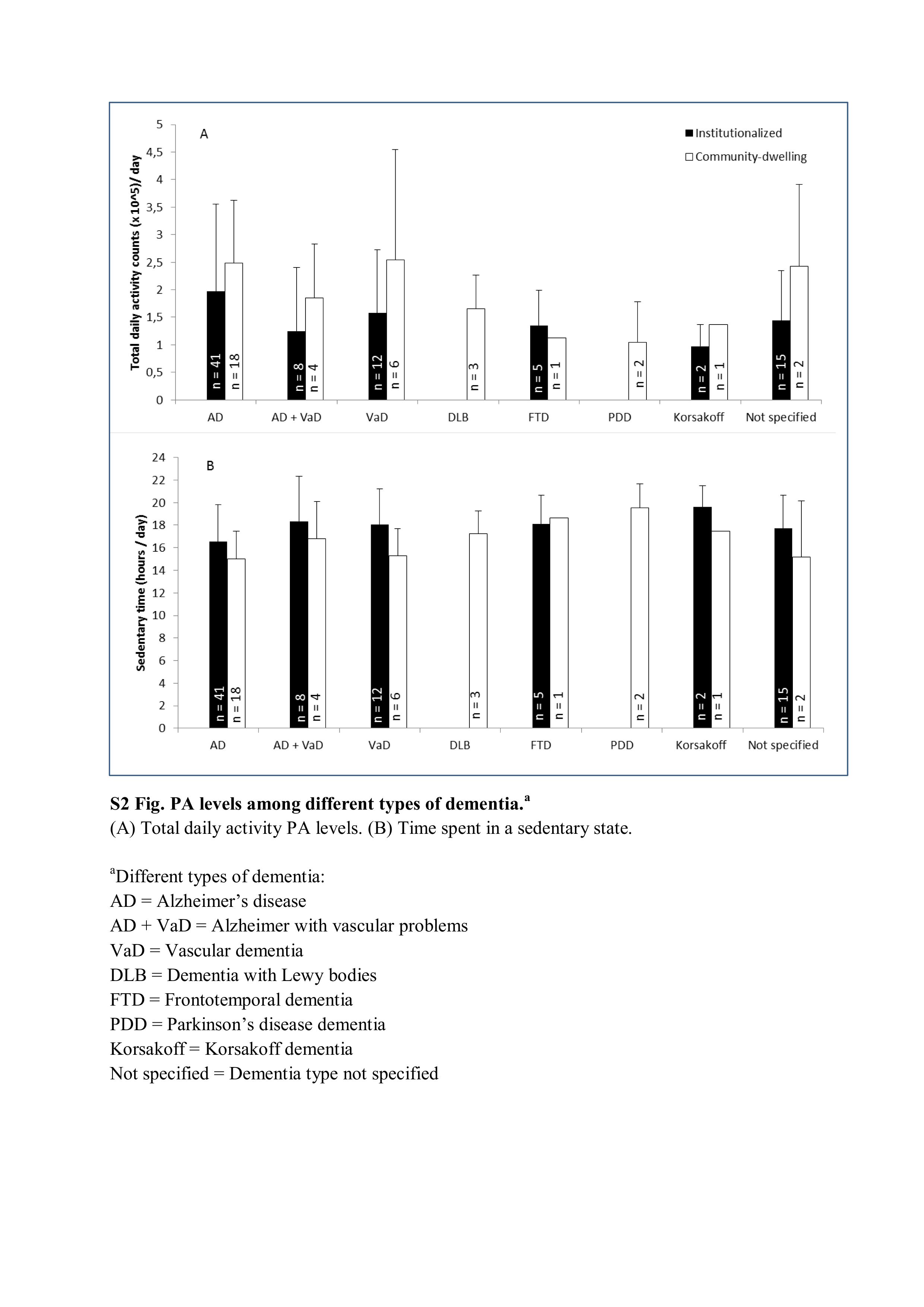

Supplement: S2 Fig — (A) Total daily PA levels. (B) Time spent in a sedentary state. (TIF) [file pone.0152457.s002.tif]
